# Harnessing the Efficiency of Twin Boron Nitride and Graphene Monolayers for Anticancer Drug Delivery: Insights from DFT

**DOI:** 10.1021/acsabm.4c01507

**Published:** 2025-02-07

**Authors:** Basant Roondhe, Rajeev Ahuja, Wei Luo

**Affiliations:** † Condensed Matter Theory Group, Materials Theory Division, Department of Physics and Astronomy, Uppsala University, Box 516, Uppsala 75120, Sweden; ‡ Department of Physics, Indian Institute of Technology Ropar, Rupnagar, Punjab 140001, India

**Keywords:** DFT study, Anticancer drug delivery, Twin monolayer, Absorption spectra, Drug release

## Abstract

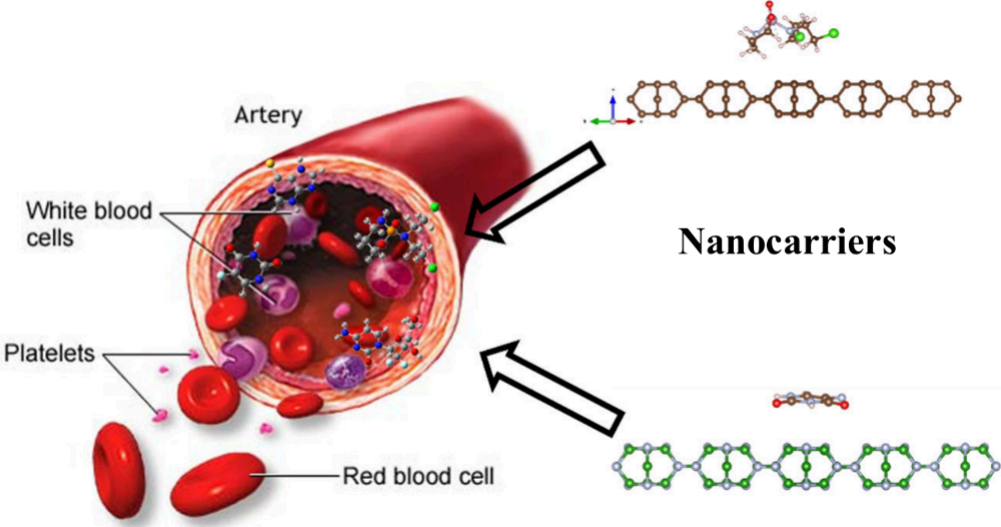

An extensive amount of research has been focused on the development of state-of-the-art methodologies for drug administration. In this study, we have utilized density functional theory (DFT) for assessing the ability of a Twin monolayer of boron nitride and graphene, i.e., Twin-BN and Twin-Gr monolayer, as a carrier for delivering four anticancer drugs (ACDs) 5-fluorouracil (5-FU), gemcitabine (GC), cyclophosphamide (CP), and mercaptopurine (6-MP). Also, the properties of all drug molecules along with the Twin-BN and Twin-Gr and the complex of the ACD-Twin-BN/Gr monolayer were investigated to explore the usefulness of the Twin-BN and Twin-Gr monolayer as ACD carrier. The interaction between the monolayers and ACDs confirmed that the adsorption is feasible as the adsorption energy ranged from −0.41 eV to −0.95 eV in the case of Twin-BN, while it ranged from −0.43 eV to −0.61 eV in the case of Twin-Gr. Additionally, the change in the band gap of the Twin-BN and Twin-Gr monolayers after the adsorption of ACDs was considerable. We can conclude that among both monolayers, Twin-BN can be utilized as a highly effective carrier for delivering ACDs. Our findings showed that the monolayer Twin-BN could be explored as a drug transporter for highly efficient carrying of the considered ACDs.

## 1 Introduction

Cancer, a group of diseases characterized by uncontrolled cell proliferation, is the predominant cause of mortality among various pathologies globally. The surge in the global population has correspondingly led to an increase in the incidence of new cancer cases. A 2019 study estimated approximately 1 335 100 new cancer diagnoses and 397 583 cancer-associated fatalities annually.^1^ A critical aspect of cancer pathogenesis is dysregulation of the cell cycle, leading to uncontrolled cell division. The disease manifests when these aberrant cells invade surrounding tissues and metastasize to distant sites within the body.^2^ Several therapeutic strategies are employed in the management of cancer including chemotherapy, radiotherapy, and surgical interventions. Chemotherapy, which utilizes a variety of pharmacological agents known as anticancer drugs, is the most commonly adopted treatment modality.^3^

Antineoplastic agents, commonly termed anticancer drugs, are pivotal in the therapeutic management of malignancies. These pharmacological compounds exhibit efficacy against neoplastic cells and are categorized into several primary classes including alkylating agents, antimetabolites, natural products, and hormones. The selection of a specific antineoplastic agent is contingent upon a multitude of factors. These encompass the type and anatomical location of the malignancy, the disease’s severity, and the potential adverse effects associated with the drug. Consequently, the choice of treatment is highly individualized, aiming to optimize therapeutic outcomes while minimizing toxicity.^4^

Numerous anticancer drugs are available, such as 6-mercaptopurine (6-MP), gemcitabine (GB), 5-fluorouracil (5-FU), and cyclophosphamide (CP). Among these, 6-MP stands out as a chemotherapy agent with immunosuppressive properties.^5^ This compound is employed in the handling of many conditions, including rheumatologic disorders, lymphoblastic diseases, cancer, and leukemia. 6-MP functions by inhibiting purine synthesis, making it particularly effective in the treatment of acute lymphoblastic leukemia.^6,7^ Given its chemotherapeutic activity, there is significant interest in the adsorption of 6-MP on nanomaterials. Since the 1950s, it has been used to treat leukemia and subsequently as an immunosuppressive and anti-inflammatory agent.^8^ Conversely, GB anticancer drug effectively treats a broad range of tumors, including those in the lungs and bladder.^9,10^ Known as a cytotoxic agent due to its cell-killing properties.^11,12^ GB has distinctive characteristics and a targeted range of effectiveness,^13,14^ which makes it a preferred option for first-line chemotherapy in patients with complexed pancreatic cancer. While cases initially respond well to GB chemotherapy, resistance can develop over time, either intrinsically or acquired. This struggle can arise from various molecular and cellular changes, such as modifications in the apoptosis pathway, alterations in nucleotide metabolism enzymes, and increased expression of drug efflux pumps.^11^

5-FU an additional powerful anticancer drug, known as a fluoropyrimidine counterpart of uracil and an essential fluorine-based agent.^15^ It works by incorporation into RNA and DNA, causing DNA damage, and inhibiting the enzyme thymidylate synthase, which is crucial for nucleotide synthesis. The fluorine atom in 5-FU mimics a hydrogen atom, converting the substrate into an inhibitor.^16,17^ Consequently, 5-FU is extensively used in the treatment of various cancers, including gastrointestinal, stomach, breast, lung, and skin cancers.^18^ Coordination chemistry indicates that chelating 5-FU with metal ions can enhance the antiproliferative effects by combining the activities of the metal and the free drug.^19^ The CP, also known as cytophosphane, is an anticancer drug used in cancer therapy and falls under the category of alkylating agents. This is employed in managing a range of cancers such as lymphatic cancer, plasma cell neoplasm, blood cancer, ovarian neoplasm, mammary cancer, oat cell carcinoma, embryonal tumor, and connective tissue cancer.^20^ It is administered as an antirejection agent for various medical conditions, including nephrotic syndrome and granulomatosis with polyangiitis, and used to manage health post-transplantation of organs. CP can be administered orally or via intravenous injection. It functions by interfering with DNA replication and RNA synthesis. Received authorization for therapeutic application within the United States during the year 1959, CP’s toxicity led to its immediate replacement with less toxic drugs when possible. Even with the apprehensions regarding its potential toxic effects, this treatment continues to be vital for serious autoimmune conditions that do not respond to disease-modifying antirheumatic medications.^20,21^

The importance of drug delivery systems has been increasingly recognized due to their integral role in the efficient transport of therapeutic agents to specific target cells.^22−24^ In an effort to mitigate adverse effects and enhance drug selectivity, researchers are exploring the use of nanomaterials as drug carriers. Nanocarriers serve as a strategic approach to address the limitations presented by anticancer medications, which include poor water solubility, notable adverse reactions, lack of targeted action, and severe toxicity.^25^ The distinct advantage of nanocarriers lies in their expansive specific surface area, facilitating the targeted administration of cancer-fighting drugs to the intended areas.^26^ A variety of nanomaterials have been employed for drug delivery applications.^27−31^ Among these, two-dimensional (2D) nanomaterials have garnered particular interest due to their advantageous properties, including a high surface-to-volume ratio, carrier mobility, solubility, and exceptional electrical and thermal conductivity. Existing research suggests that 2D materials based on boron nitride and graphene exhibits promising biocompatibility profiles.^23,24^ Preliminary studies have indicated that these nanostructures generally show low cytotoxicity and good biocompatibility, making them suitable candidates for biomedical applications including drug delivery systems. Research has confirmed the critical importance of surface chemistry in nanomaterials for ensuring their compatibility with biological systems and controlling drug actions. This understanding underscores the potential of nanomaterials in the development of more effective and safer drug delivery systems.

**Figure 1:**
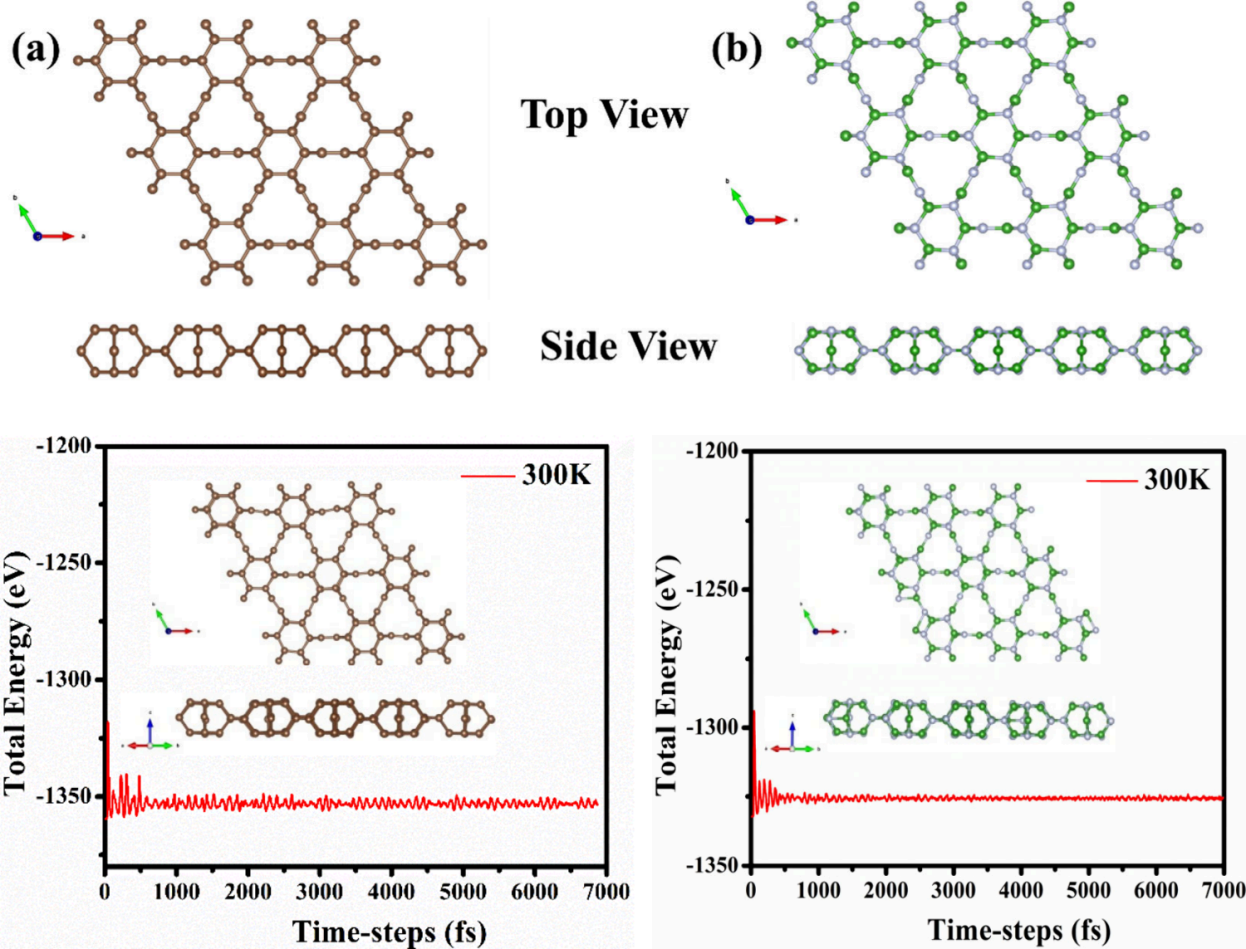
Structure of monolayer from the top view and side view along with the AIMD Molecular dynamics simulation showing the total energy of the both sheet at 300 K with a 1 fs time step for a 7 ps time period (a) Twin-Gr and (b) Twin-BN. The C, B, and N atoms are colored in brown, green, and gray balls, respectively

Recently, a novel two-dimensional (2D) allotrope of carbon, termed “twin graphene”, has been predicted.^32^ Twin graphene, akin to a bilayer graphene, comprises two organically conjoined layers. This unique structure is achieved by substituting one-third of the parallel aromatic bonds in AA-stacked bilayer graphene with carbon dimers (Figure 1a). From a top view, the structure of twin graphene bears a resemblance to γ-graphyne. Earlier research has indicated that the phonon spectrum in twin graphene lacks any imaginary frequencies,^32^ confirming its dynamic stability. The semiconducting properties of twin graphenes suggest potential widespread applications in future semiconductor-based nanoelectronic devices. Mirroring the properties of Twin-Gr, a distinct boron nitride configuration known as “Twin BN” is created by fusing two boron nitride layers in an AA stacking arrangement. This structure bears resemblance to the carbon-based material analyzed by Jiang et al.^32^ By replacing a third of the parallel aromatic bonds in the AA-stacked bilayer graphene with a carbon–carbon dimer, a feature Twin BN also possesses, it is expected to have an impact on its electronic properties. Twin BN, inspired by the adaptability and modifiability seen in analogous carbon structures, is distinguished by its extensive conjugated π-electronic structures, a multitude of chemical bonds, and stability. The configuration is derived from substituting one-third of the parallel aromatic linkages in AA-aligned dual-layer boron nitride and graphene with dimer formations. These single layers are akin to a bilayer of graphene and boron nitride, where one surface is gradually fused to the other, leading to the terms Twin-BN and Twin-Gr.

Further, the absence of detailed molecular insight into the interactions at play on anticancer drugs with the 2D material motivates us to explore Twin monolayers as a drug carrier. In this study, we have explored two novel 2D planar direct semiconducting Twin monolayers of boron nitride and graphyne, i.e., Twin-BN and Twin-Gr, based on first-principles calculations. Our calculations reveal that both are direct bandgap semiconductors. The value of the bandgap is on par with that of key semiconducting materials used in industry, which positions them as potential contenders for the next generation of drug delivery materials.

## 2 Computational Method

In present study, we examined the geometrical, electronic, and electrochemical characteristics using density functional theory (DFT) within the scope of the Vienna Ab Initio Simulation Package (VASP).^33^ We applied the generalized gradient approximation as described by Perdew, Burke, and Ernzerhof (GGA-PBE) self-consistently.^34^ This was done within the context of the projector augmented wave (PAW) method and a plane-wave basis set. A kinetic energy cutoff of 500 eV was established as the standard for all subsequent calculations, adhering to convergence thresholds. During the optimization of the geometry, the convergence parameters were meticulously set to 10^–6^ eV for energy and 10^–3^ eV/Å for force. In the process of optimizing the geometry for all configurations, we established a vacuum spacing of 15 Å in the *z*-direction to prevent interactions between the layered structures and their periodic counterparts. For precise property determination, a comprehensive Monkhorst Pack K-point grid of approximately 8 × 8 × 1 was utilized in reciprocal space during these optimizations,^35^ as indicated by the convergence test of total energy versus K-points. The interaction of the drug molecule with the surface was assessed using the Bader charge analysis method.^36^ Recognizing that the GGA-PBE approach tends to undervalue binding energies, especially those influenced by van der Waals forces, we opted for the DFT-D3 technique proposed by Grimme,^37^ as incorporated in the VASP, to account for the van der Waals adjustments. In the present study, we have employed Grimme’s dispersion correction (DFT-D3), which includes both pairwise terms and three-body dispersion terms, offering a more accurate and comprehensive description of dispersion interactions. The pairwise term *C*_6*ij*_/*R*_*ij*_^6^ considers the long-range behavior between atoms, where *C*_6*ij*_ is the dispersion coefficient, and *R*_*ij*_^6^ is the distance between the atoms. Additionally, DFT-D3 accounts for three-body interactions, improving the accuracy and reliability of the calculations. DFT-D3 effectively explains the noncovalent, long-distance van der Waals (vdW) interactions between the adsorbent and the adsorbate. This method has been chosen for our calculations, as it provides a precise and reliable depiction of noncovalent interactions, ensuring the accuracy of the results.

Compared to not using van der Waals corrections, incorporating DFT-D3 significantly improves the accuracy of modeling noncovalent interactions. Without these corrections, the calculations can underestimate the binding energies and fail to capture long-range interactions accurately. This can lead to incorrect predictions of the interaction energies. For comparison, we have calculated the adsorption energy with and without the dispersion correction and provided these results in Table S1 of the Supporting Information. This allows for a clear demonstration of the impact of including van der Waals interactions in our calculations.

Ab initio molecular dynamics (AIMD) simulation within Nose–Hoover thermostat is performed in order to see the thermal stability of both the twin sheets.^38^ The absorption energy calculations were based on the specific interaction between the anticancer drug molecules and the Twin monolayer:
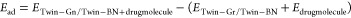
1*E*_Twin–Gn/Twin–BN+drug molecule_ signifies the total energy derived from DFT calculations, reflecting the interaction of adsorbents with Twin-Gr and Twin-BN sheets. These adsorbents include 6-mercaptopurine (6-MP), gemcitabine (GB), 5-fluorouracil (5-FU), and cyclophosphamide (CP). *E*_Twin–Gr/Twin–BN_ is the total energy of pristine Twin-Gr/Twin-BN sheet and *E*_drug molecule_ represents the energy of individual adsorbents. It is crucial to note that negative values of *E*_ad_ signify a favorable binding interaction between the adsorbents, while positive values indicate a less favorable binding interaction.

## 3 Results and Discussion

### 3.1 Structural and Electronic Properties of Pristine Twin-Gr and Twin-BN

To understand the structural and electronic characteristics of a system comprehensively, it is crucial for the system to be in a state of complete relaxation and optimization. The initial step involved fine-tuning the geometry and relevant parameters of the basic units of both Twin-GR and Twin-BN sheet configurations, which was accomplished by determining their lattice constants and ground energy levels. The optimized structures of the Twin-GR and Twin-BN monolayers are depicted in Figure 1. Both Twin-Gr and Twin-BN possess a space group of *P*6/*mmm* in Hermann-Mauguin notation, which is symmorphic and belongs to the *D*_6*h*_ point group. In Twin-Gr, there are two types of inequivalent carbon atoms, denoted as C1 (located on the surface planes) and C2 (situated on the midplane), respectively (Figure 1a). The Twin-Boron Nitride (Twin-BN) framework is characterized by two unique B–N bond types, labeled as (B–N)_1_ (present on the exterior layers) and (B–N)_2_ (situated in the central layer), as depicted in Figure 1(b). Each structure’s primitive unit cell, is composed of 18 atoms. Specifically, the Twin-Graphene (Twin-Gr) structure is made up of 12 C1 atoms and 6 C2 atoms, totaling C18, whereas the Twin-BN structure is an assembly of 9 boron (B) and 9 nitrogen (N) atoms. It is interesting to note that when viewed from above, both structures exhibit a configuration that is strikingly similar to that of gamma-graphyne and boron nitride variants that mimic graphyne.^32,39,40^ The optimized structural parameters are summarized in Table 1. With regard to the thermal stability of both Twin monolayers, we performed AIMD with a time step of 1.0 fs at 300 K using a 3 × 3 supercell. There is no structural deformation in both monolayers; furthermore, the oscillation range for the total energy against the time step is provided as displayed in Figure 1.

**Table 1:** Calculated Adsorption Energy (*E*_ad_, Charge Transfer (*Q*)^a^, and Minimum Interaction Distance (*d*), Band Gap, Change in Band Gap, Work Function, Change in Work Function

System	*E*_ad_ (eV)	*Q* (e)	*d* (Å)	*E*_g_ (eV)	Δ*E*_g_ (eV)	Φ (eV)	ΔΦ (eV)
**Twin-BN**				**3.316**		**6.38**	
**Twin-BN + 5-FU**	**–0.41**	**↑ 0.014**	**2.83**	**3.004**	**0.312**	**5.97**	**0.41**
**Twin-BN + CP**	**–0.64**	**↑ 0.026**	**2.61**	**2.399**	**0.917**	**5.66**	**0.72**
**Twin-BN + GB**	**–0.58**	**↑ 0.036**	**2.74**	**2.374**	**0.942**	**5.68**	**0.70**
**Twin-BN + 6-MP**	**–0.95**	**↓ 0.241**	**2.09**	**1.615**	**1.701**	**5.20**	**1.18**
**Twin-Gr**				**0.725**		**5.79**	
**Twin-Gr + 5-FU**	**–0.43**	**↑ 0.017**	**4.33**	**0.714**	**0.011**	**5.80**	**0.01**
**Twin-Gr + CP**	**–0.60**	**↑ 0.031**	**2.51**	**0.415**	**0.310**	**5.67**	**0.12**
**Twin-Gr + GB**	**–0.58**	**↑ 0.033**	**2.61**	**0.373**	**0.352**	**5.64**	**0.15**
**Twin-Gr + 6-MP**	**–0.61**	**↓ 0.145**	**4.30**	**0.28**	**0.445**	**5.33**	**0.46**

a **↑** indicates charge toward molecule away from the surface and **↓** indicates charge toward the surface.

The distance between two C1 atoms (denoted as *r*_11_ = 1.42 Å) suggests that the C1 atoms are sp^2^ hybridized. Meanwhile, the bond lengths between C1 and C2 (notated as *r*_12_ = 1.55 Å) and between two C2 atoms (notated as *r*_22_ = 1.34 Å) demonstrate the presence of single and double bonds in the Twin-Gr structure, respectively. For the Twin-BN structure, the bond length between B1 and N1 (notated as *r*_11_ = 1.45 Å) is indicative of sp^2^ hybridization. The bond lengths between B1 and N2 (notated as *r*_12_ = 1.52 Å) and between B2 and N2 (notated as *r*_22_ = 1.39 Å) reveal distinct single and double bond characteristics, respectively. The bond lengths we observed are consistent with those reported in prior studies.^39^ Dynamic stability of the Twin-Gr and Twin-BN monolayers has been reported in the literature.^39,40^

**Figure 2:**
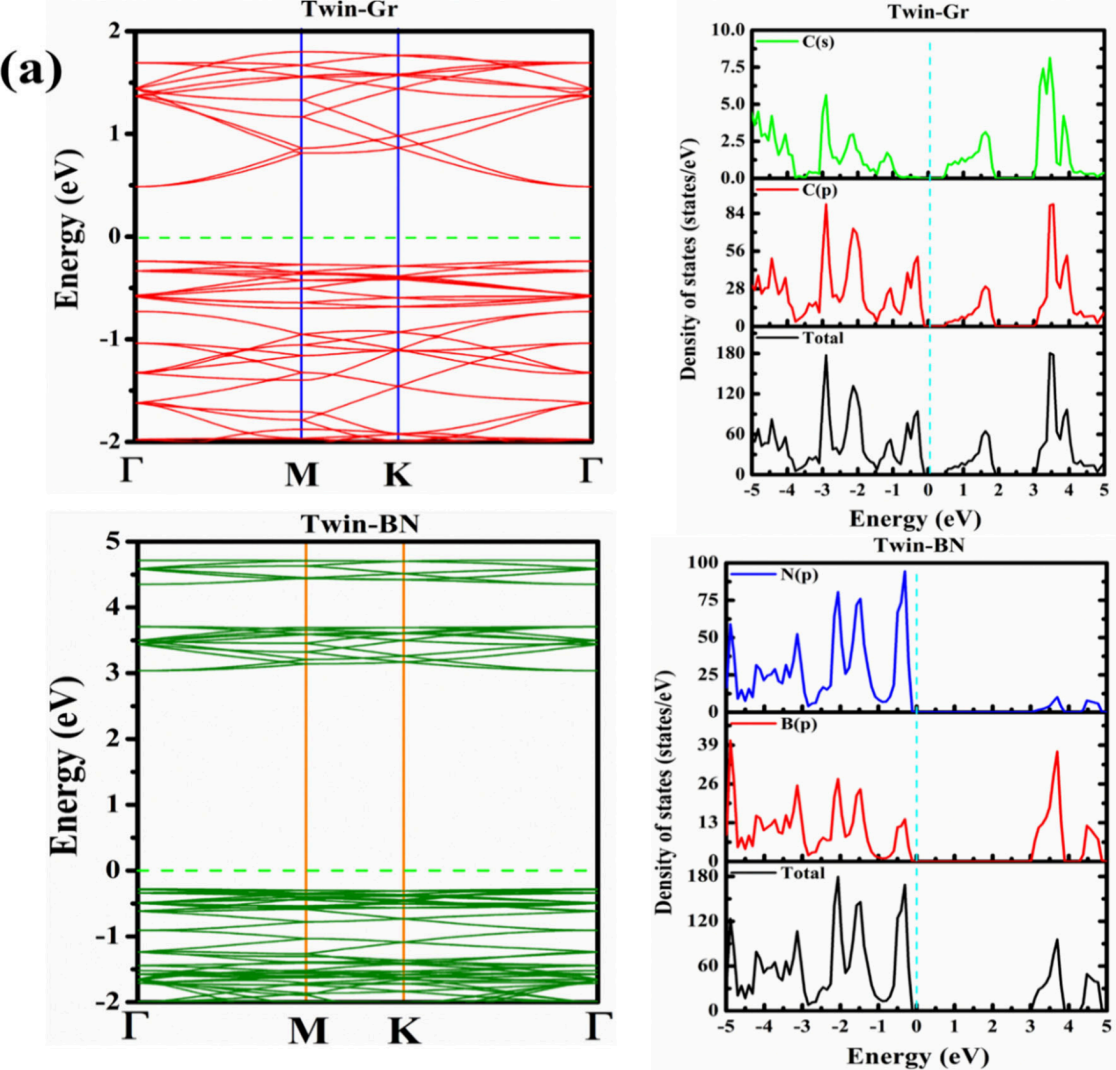
Electronic band structure along with the total and partial DOS plots of pristine (a) Twin-Gr and (b) Twin-BN. The Fermi level is set to 0 eV.

Recognizing the pivotal role of the electronic band structure in deciphering a material’s electronic trait, we proceeded to compute the band structures for the monolayers of Twin-Gr and Twin-BN. Insights into the electron mass are gleaned from the band dispersion analysis. The band structures for both Twin-Gr and Twin-BN, mapped along the k-path Γ–M–K−Γ within the Brillouin Zone (BZ), are depicted in Figure 2. We have also calculated the band structures of Twin-Gr and Twin-BN using the HSE06 functional, and the results are provided in Figure S1 in the Supporting Information. This illustration is complemented by the density of states and the orbital contributions, represented as the Projected Density of States (PDOS). The conduction bands are highly dispersive and hint at a diminutive effective electron mass. Notably, a direct band gap is discerned at the Γ point, 0.7 eV for Twin-Gr and 3.31 eV for Twin-BN corroborating findings from earlier research.^39−44^ The calculated HSE06 band gap value for Twin-Gr is 1.5 and 4.73 eV for Twin-BN (see Figure S1). The notably modest and direct band gaps of both Twin-Gr and Twin-BN underscore their potential for applications in drug delivery and biosensor technologies.^28,30,31^ In the Twin-Gr structure, the s and p orbitals of carbon atoms contribute equally to both the conduction band (CB) and the valence band (VB). Conversely, in the Twin-BN structure, boron and nitrogen atoms jointly influence the formation of the electronic band structure. Unlike boron, which impacts both the CB and the VB, nitrogen influence is predominantly in the CB. Figure 2 (right panel) elucidates the total and partial densities of states for Twin-Gr and Twin-BN, shedding light on the electronic involvement.

### 3.2 Adsorption Mechanisms

**Figure 3:**
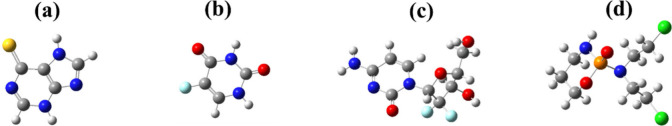
Optimized structure of anticancer drugs (a) 6-MP, (b) 5-FU, (c) GB, and (d) CP. The C, N, S, F, Cl, H, P, and O atoms are colored in gray, blue, yellow, cyan, green, white, orange, and red balls, respectively.

To grasp the underlying processes governing the interactions among monolayers (Twin-Gr and Twin-BN) and anticancer drug, a supercell is made (3 × 3) of Twin monolayer with 162 atoms (162 C atoms in Twin-Gr and 81 B and 81 N atoms in Twin-BN) to provide a large surface for drug molecule to interact. The full structural optimization of Twin monolayer sheet (Twin-Gr and Twin-BN) and all considered anticancer drugs (ACDs) molecules 5-fluorouracil (5-FU), gemcitabine (GC), cyclophosphamide (CP), and 6-mercaptopurine (6-MP) was conducted individually. The optimized structures of ACDs molecules are presented in Figure 3(a–d).

**Figure 4:**
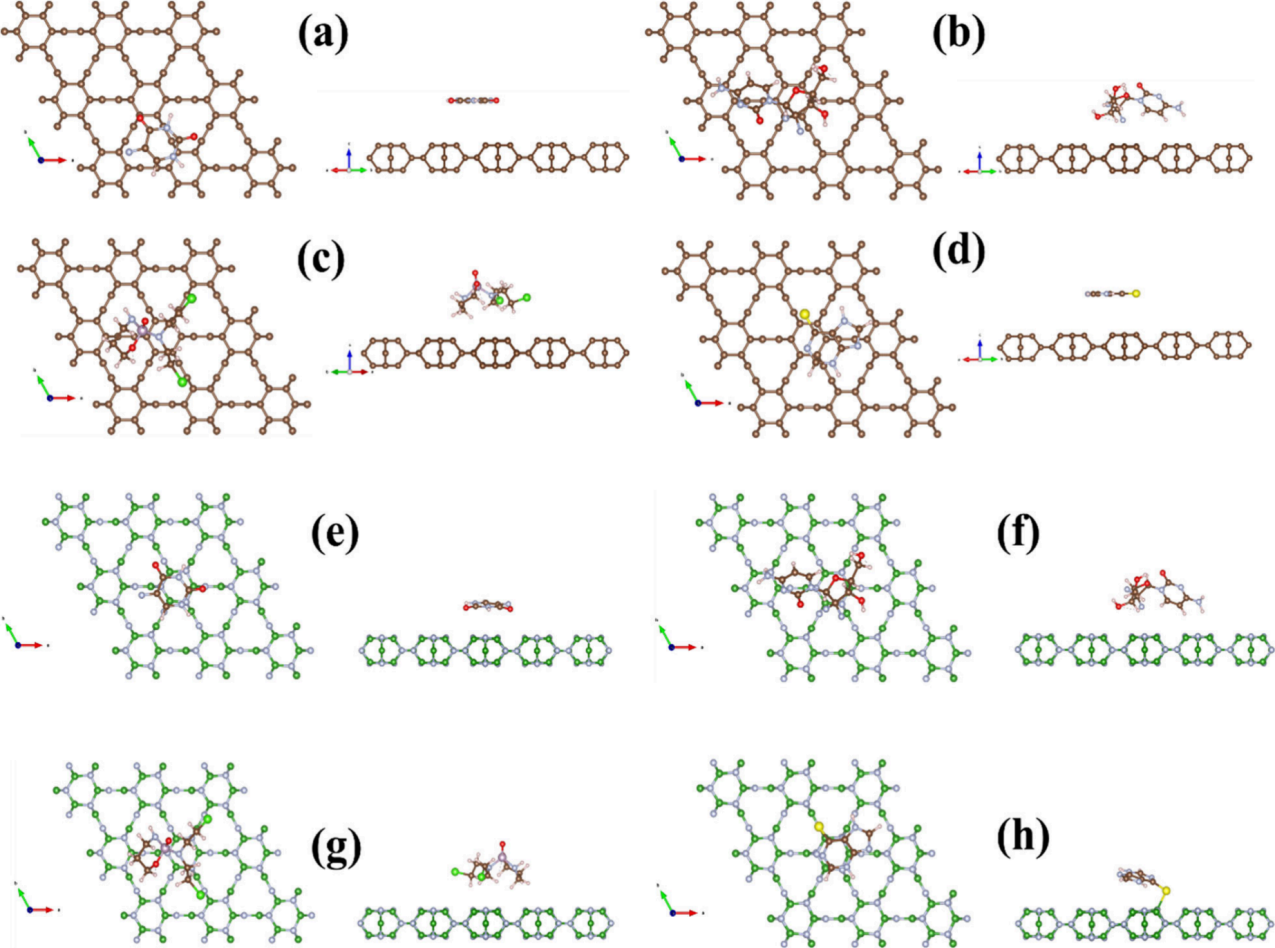
Optimized structure of anticancer drugs adsorbed monolayers: (a) 5-FU@Twin-Gr, (b) GB@Twin-Gr, (c) CP@Twin-Gr, (d) 6-MP@Twin-Gr, (e) 5-FU@Twin-BN, (f) GB@Twin-BN, (g) CP@Twin-BN, and (h) 6-MP@Twin-BN.

Figure 4(a–h) show the optimized geometries of Twin-Gr and Twin-BN sheet with adsorbed ACDs molecules. For all ACDs molecules arrayed over the Twin-Gr and Twin-BN sheets, a parallel alignment was selected, echoing prior studies that indicate this arrangement as the most stable form.^27−31^ The process of structural relaxation commenced with the initial placement of the molecule in the proximity of the monolayer surface, which was then followed by a comprehensive structural relaxation to ascertain the most stable geometric configuration. In the final stable configurations across all systems under study, the molecules maintained a separation ranging from 2.09 to 4.33 Å from the Twin monolayer surface. This considerable separation precludes the possibility of chemical bond formation with the Twin monolayer, resulting in the physisorption of the molecules, a finding that is consistent with our complete structural relaxation calculations.

In every instance except for 6-MP on Twin-BN, the adsorption process is of a physical nature, known as physisorption. This is due to the substantial separation between the 6-MP and Twin-BN sheets, approximately 2.09 Å, as indicated in Table 1. Such a significant gap between the ACDs and the Twin sheets effectively rules out the formation of covalent bonds. We have calculated the adsorption energy with and without the incorporation of vdW correction and the comparative data is shown in Table S1 in the Supporting Information. Significant increase in the adsorption is observed after the incorporation of the DFT-D3 correction (see Table S1). The *E*_ad_ calculated for 6-MP, GB, 5-FU, and CP over Twin-BN are −0.95, −0.58, −0.41, and–0.64 eV, while the values are −0.61, −0.58, −0.43, and–0.60 eV over Twin-Gr, respectively, with binding distances (*d*) between 2.09 and 4.33 Å. The sequence of adsorption of the ACDs over Twin-BN is 6-MP > CP > GB > 5-FU, which is similar to that of over Twin-Gr (6-MP > CP > GB > 5-FU). The configuration involving π–π stacking exhibits a greater adsorption energy because it reduces the repulsion that arises from the overlapping of π-orbitals. This reduction in repulsion helps in preventing the molecules from being pushed away from the nanosurface, thereby resulting in enhanced *E*_ad_ on the nanosurface.^28−30^

**Figure 5:**
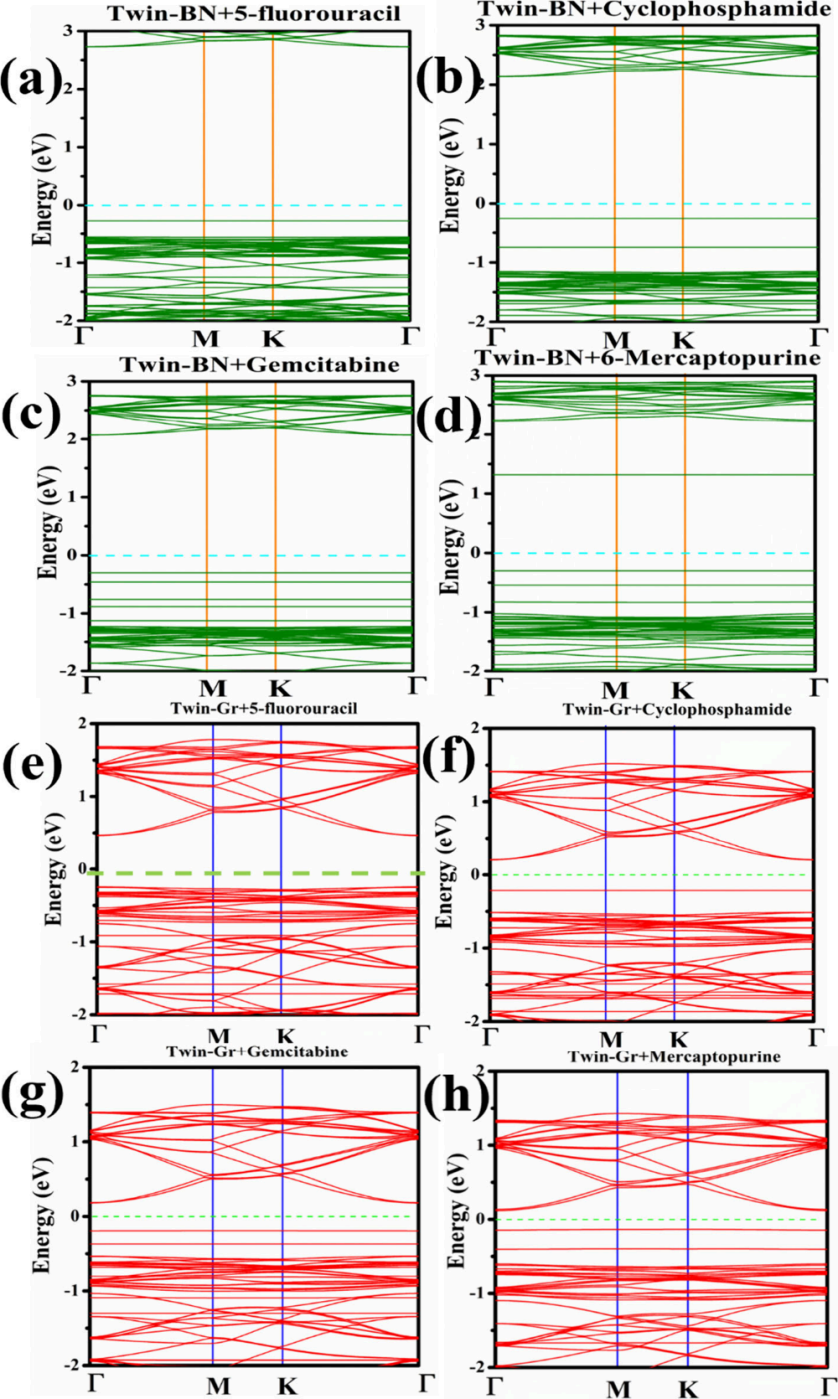
Band structures of anticancer drugs adsorbed monolayers: (a) 5-FU@Twin-BN, (b) CP@Twin-BN, (c) GB@Twin-BN, and (d) 6-MP@Twin-BN. (e) 5-FU@Twin-Gr, (f) CP@Twin-Gr, (g) GB@Twin-Gr, (h) 6-MP@Twin-Gr

The binding of ACD molecules to Twin sheets results in modifications to the electronic characteristics, which were analyzed through the evaluation of band dispersion, band gap, and the density of states (DOS), including the partial DOS (PDOS). The graphical representation of the band dispersion for ACD-bound Twin sheets is showcased in Figure 5. Notable alterations in both the band structure and the band gap are evident from the dispersion analysis. Postadsorption, the band structure reveals the emergence of impurity states, predominantly attributed to the p-orbital electrons of carbon, oxygen, and nitrogen atoms, with a supplementary role played by the *s* orbital electrons of hydrogen atoms within the ACD. Additionally, the formation of distinct flat bands within both the conduction and valence band regions of the band structure is a noteworthy observation. This phenomenon can be linked to the suppression of electron kinetic energy.^28^ These flat bands arise from the involvement of the *p*_*x*_ orbital electrons of the nitrogen and carbon atoms. This pattern of band behavior, characterized by the presence of numerous flat bands, is consistent across all ACD molecules and is discernible in both the valence and conduction bands.

The band gap of Twin-BN decreases from 3.316 to 3.004, 2.399, 2.374, and 1.615 eV after the adsorption of 5-FU, CP, GB, and 6-MP, respectively. While in case of Twin-Gr the reduction of band gap from 0.725 to 0.714, 0.415, 0.373, and 0.28 after the adsorption of 5-FU, CP, GB, and 6-MP, respectively. Our subsequent examination involved assessing the charge redistribution through Bader charge analysis. The pronounced charge displacement observed between the ACD molecules and the Twin monolayers suggests that the ACD interaction could result in alterations to the electronic characteristics of both the Twin-Boron Nitride and Twin-Graphene monolayers, as detailed in Table 1. Such interactions are likely contributors to the observed modulation of the bandgap.

**Figure 6:**
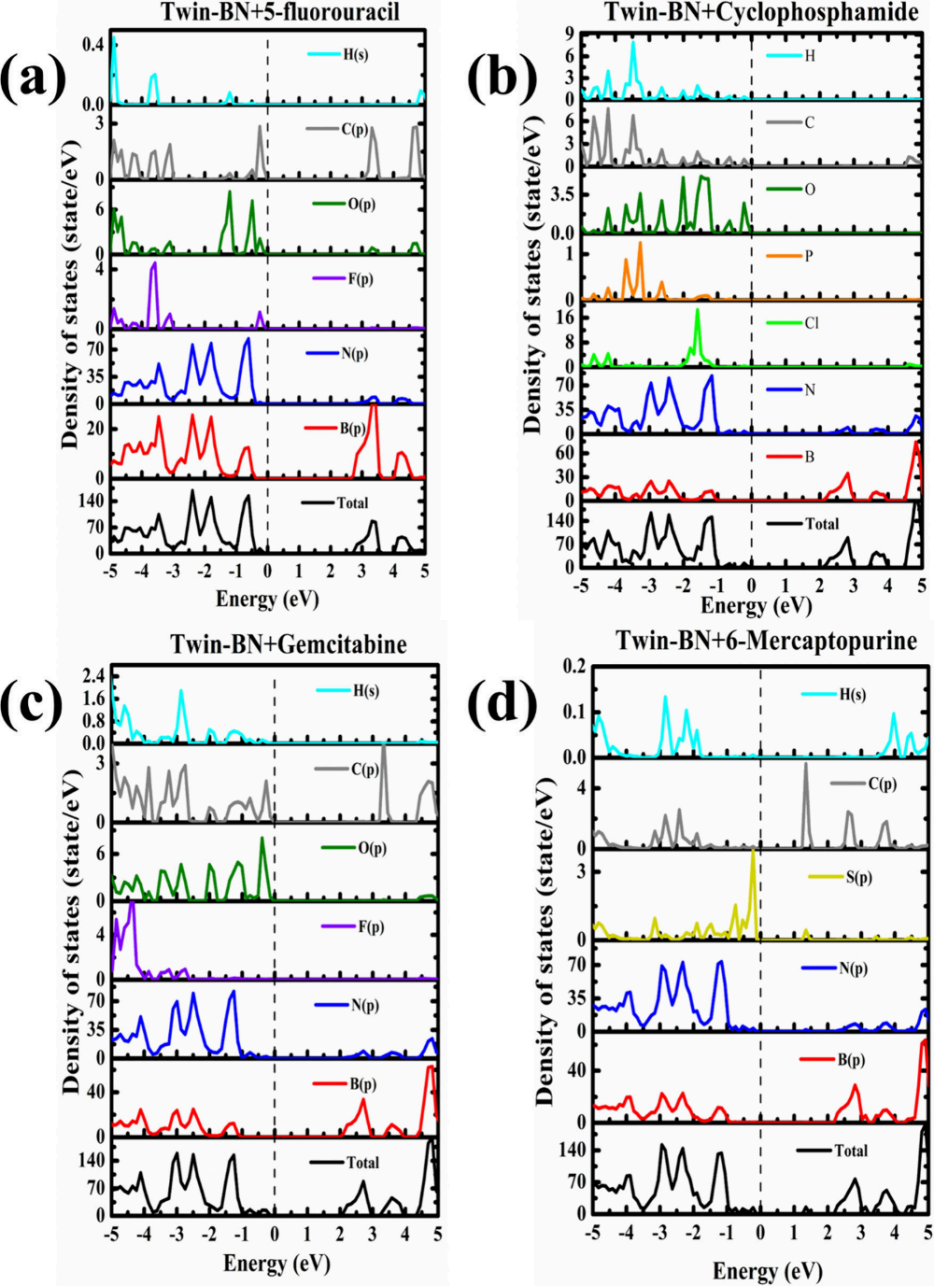
Total and partial DOS of anticancer drugs adsorbed Twin-BN monolayers: (a) 5-FU@Twin-BN, (b) CP@Twin-BN, (c) GB@Twin-BN, and (d) 6-MP@Twin-BN.

**Figure 7:**
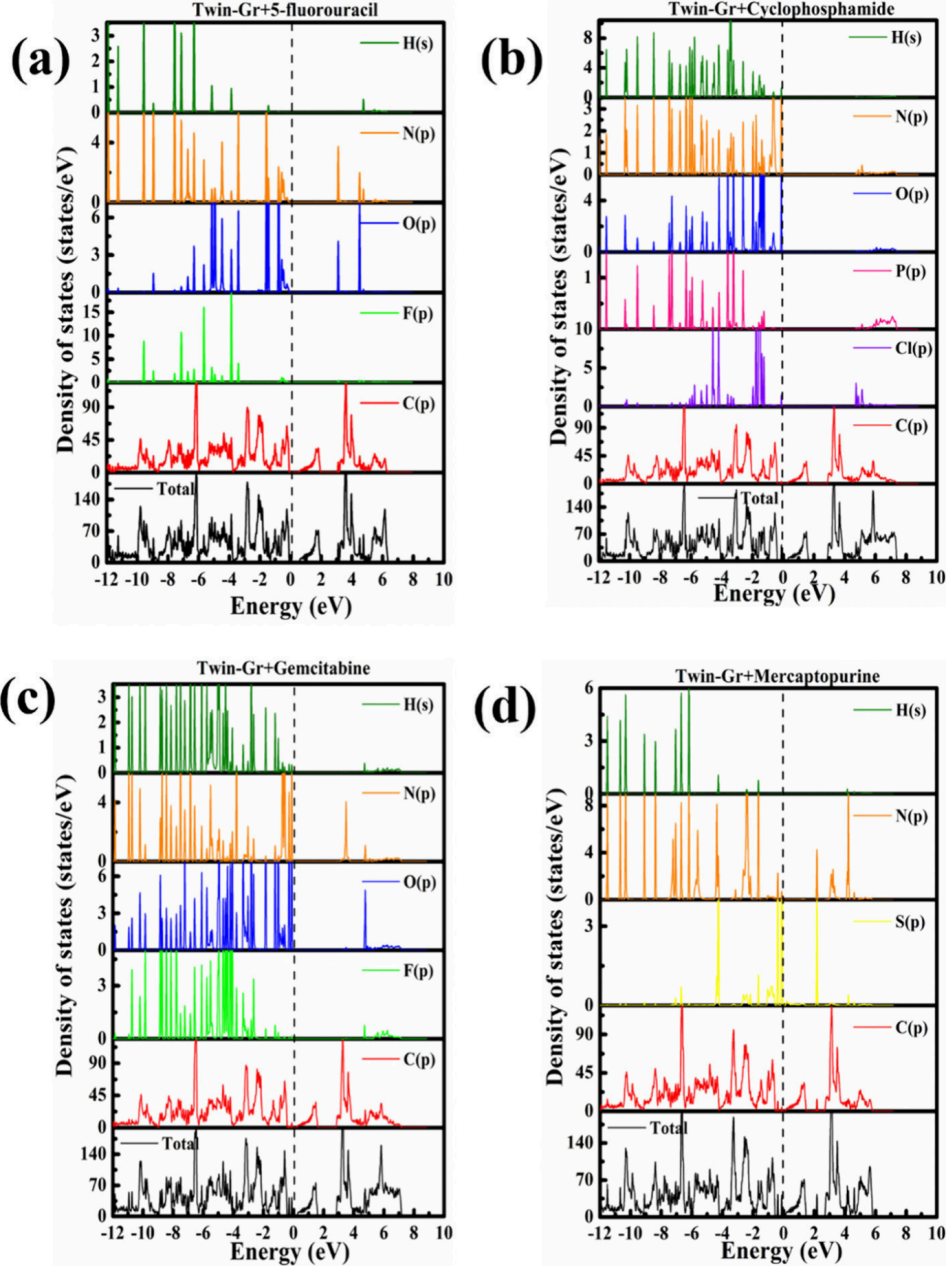
Total and partial DOS of anticancer drugs adsorbed Twin-Gr monolayers: (a) 5-FU@Twin-Gr, (b) CP@Twin-Gr, (c) GB@Twin-Gr, and (d) 6-MP@Twin-Gr

To shed light on the molecular impact on electronic characteristics, we performed calculations of the DOS and the PDOS for ACD molecules attached to Twin-Boron Nitride, as depicted in Figure 6, and those bound to Twin-Gr, as shown in Figure 7. The primary benefit of these DOS and PDOS calculations lies in their ability to facilitate a straightforward assessment of the electronic state contributions from the adsorbed molecules. Figures 6 and 7 demonstrate that the interaction between the Twin sheets and the ACD molecules leads to the emergence of modest peaks within the valence band area close to the Fermi level. The plots from the HSE06 DOS calculations are provided in the Figure S2 and S3 of Supporting Information for Twin-BN and Twin-Gr with ACD molecules. The band gap values increase in case of HSE06 calculations compare to the PBE method which suggests the correctness of our calculations. The DOS data unequivocally indicates that the ACD molecules induce substantial changes to the electronic properties of the Twin sheets, particularly in the lower valence band region where the interaction spawn impurity states. The predominant influence stems from the *p* orbital electrons, responsible for the additional peaks. Specifically, 6-MP significantly alters the band gap of the pristine Twin sheets due to the introduction of supplementary electronic states, a consequence of the sulfur atom presence.

The decrease in the band gap upon the adsorption of molecules is primarily due to the interaction and hybridization of electronic states between the adsorbate and substrate, which leads to the formation of new hybridized states that reduce the energy difference between the conduction and valence bands. Additionally, charge transfer between the adsorbate and substrate shifts the energy levels and affects the density of states near the Fermi level. Adsorption introduces new surface states leading to states within the original band gap, thereby narrowing it. Moreover, adsorption can distort the atomic positions and alter the electronic band structure, contributing to a decrease in the band gap. These combined effects result in the commonly observed reduction in the band gap upon molecular adsorption.

The magnitude of the energy gap (*E*_g_) plays a crucial role in determining the electrical conductivity (σ) of materials. This is due to the fact that the energy necessary to liberate an electron from the valence band to transform into a mobile charge carrier corresponds to the bandgap. The traditional correlation between *E*_g_ and σ of a substance is encapsulated by the subsequent equation:^28,45^
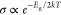
2where *k* and *T* are the Boltzmann’s constant and the temperature, respectively. Derived from eq 2, there is an inverse relationship between conductivity and the bandgap, suggesting that a smaller bandgap *E*_g_ leads to greater electrical conductivity at a specific temperature *T*. Therefore, when ACD molecules are adsorbed onto Twin surfaces, the observed reduction in the band gap correlates with an enhanced σ for both types of sheets. As a result of the ACD molecules’ adsorption, the energy gap is modified, becoming more constricted. This, in turn, boosts the conductivity of the nanosheet, which serves as evidence of the robust interaction between the molecule and the Twin sheets. The alteration in the Fermi level of Twin sheets caused by the ACD molecules generates an electrical signal, which could render them suitable for use in biosensor applications.

**Figure 8:**
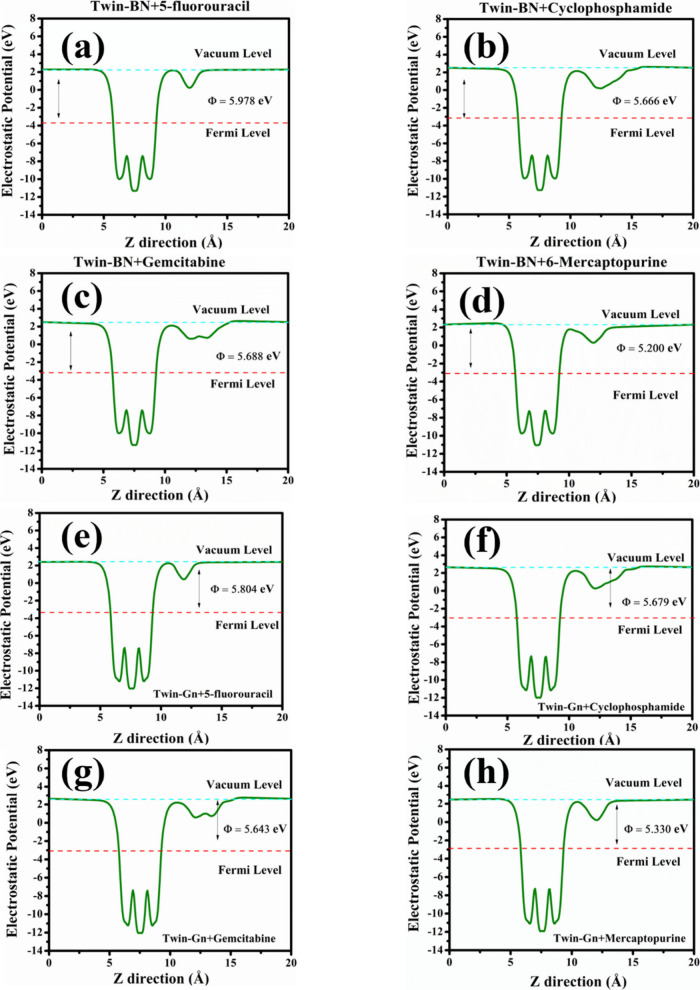
Work function plots of anticancer drugs adsorbed monolayers (a) 5-FU@Twin-BN, (b) CP@Twin-BN, (c) GB@Twin-BN, (d) 6-MP@Twin-BN, (e) 5-FU@Twin-Gr, (f) CP@Twin-Gr, (g) GB@Twin-Gr, and (h) 6-MP@Twin-Gr.

We have also determined the work function of both unaltered and ACD molecule-coated Twin sheets to assess their potential as sensors. The work function, denoted as ϕ, represents the energy needed to extract an electron from the most occupied state within the Fermi distribution of a material to a position just beyond the solid surface in a vacuum at zero Kelvin.
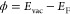
3*E*_vac_ represents the vacuum level energy, while *E*_F_ stands for the Fermi level energy. The Fermi energy level was ascertained by integrating the DOS from the lowest energy up to a level that accounts for the total electron count in a unit cell. *E*_vac_ was determined through the planar average of electrostatic potential energy in the *z*-axis, which is oriented toward the vacuum. The work function plots are illustrated in Figure 8. On these plots, two negative peaks are noticeable within the electrostatic potential curves, with the pronounced and subdued peaks corresponding to the Twin nanosheet and the ACD molecule, respectively. Postadsorption, the work function for both Twin-BN and Twin-Gr exhibits a marked alteration, as seen in Table 1. This shift in the work function confirms the occurrence of physical adsorption of ACD molecules onto the surface of both materials, driven by substantial interactions. Our findings, which display a modulation in the electronic properties following adsorption, point to the feasibility of detecting ACD molecules using both Twin-BN and Twin-Gr sheets. The Twin BN and Twin-Gr have advantages over traditional graphene and h-BN. Both Twin BN and Twin-Gr exhibit unique semiconducting properties due to their altered band structures, achieved by substituting one-third of the parallel aromatic bonds with carbon dimers. This tunability allows for better control over the electronic properties, enhancing efficiency in electronic and optoelectronic devices compared to the more rigid structures of pure graphene and h-BN. Furthermore, the unique structure of Twin-Gr and Twin BN, which includes organically conjoined layers and extensive conjugated π-electronic structures, provides a higher surface area compared to traditional graphene and hBN. This increased surface area is particularly beneficial for applications such as drug delivery and adsorption, where a larger surface area improves interaction efficiency with other molecules. Additionally, the extensive conjugated π-electronic networks in twin structures offer enhanced chemical interactions and functionalization potential, making these materials more adaptable to various applications.

### 3.3 Optical Absorption

**Figure 9:**
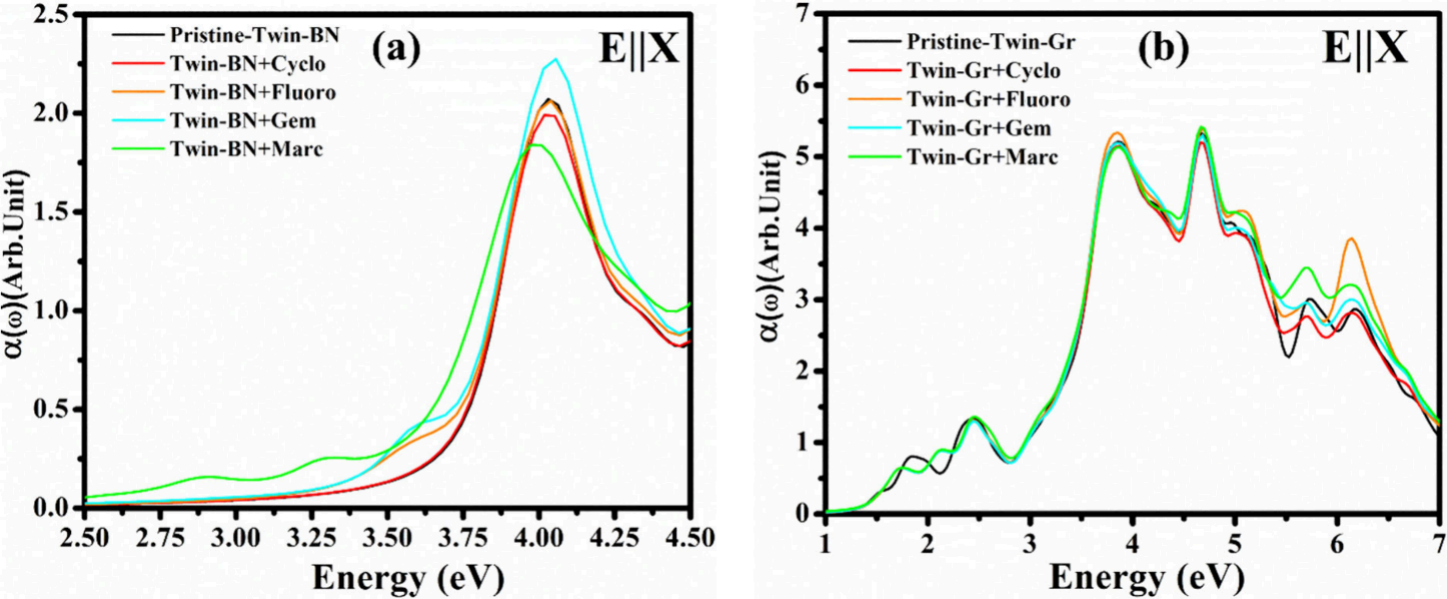
Absorption plots of anticancer drugs adsorbed monolayers: (a) Twin-BN and (b) Twin-Gr.

For the computation of optical characteristics, we employed Density Functional Theory (DFT) within the framework of the Random Phase Approximation (RPA),^46^ where the local field effects are accounted for solely at the Hartree level. The calculation of the frequency-dependent dielectric matrix by VASP is conducted subsequent to the establishment of the electronic ground state. The dielectric function is expressed as a combination of its real and imaginary components, denoted as ε(ω) = ε_1_(ω) + iε_2_(ω). Here, imaginary component ε_2_(ω) is derived directly from the electronic band structure through the use of optical matrix elements. In order to compute the real component ε_1_(ω), the Kramers–Kronig relations were utilized.^47,48^
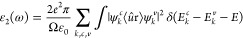
4
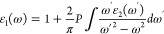
5The absorption coefficient was determined by using the following equation:
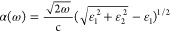
6Recent times have witnessed a marked increase in the exploration of optical sensors. Both experimental and theoretical research suggest that two-dimensional materials hold significant promise for deployment in optical sensing devices.^49^ In pursuit of understanding the prospective uses of Twin BN and Twin Gr monolayers as materials sensitive to optical stimuli, we calculated the impact on the optical characteristics of both unmodified Twin BN and Twin Gr monolayers as well as those with adsorbed ACD molecules. The adsorption of ACD’s molecules shows significant alteration in the absorption coefficients (Figure 9). From graph we can get an idea that in both Twin monolayer absorptions happen in the visible as well as in the UV region, which suggests that both the considered monolayer can have considerable potential in photoelectric devices particularly in the between visible and UV area. This situates the threshold of absorption within the spectrum of visible light, which is a factor that is essential for the efficacy of photocatalytic processes in real-world applications. Our absorption results confirm the similar trend which we have obtained with electronic properties, adsorption energy, and work function calculations. Our comparative analysis of ACDs on Twin monolayers indicates that Twin-BN demonstrates a more pronounced interaction with all ACDs in contrast to Twin-Gr, likely due to its ionic properties. As far as we are aware, the application of Twin monolayers as carriers for ACDs has not yet been realized in practical experiments. It is our aspiration that future endeavors will focus on the creation and evaluation of the practicality of biosensors and drug delivery systems based on Twin-BN and Twin-Gr. We are optimistic that our research will pave the way for experimentalists to forge ahead in the development of anticancer drug carriers utilizing Twin monolayer technology.

### 3.4 Drug Release

The mechanism of drug release plays a crucial role in the efficacy of drug-delivery systems. For this study, we determined the timeframes for drug desorption from the Twin sheets by applying the principles of the van’t Hoff–Arrhenius equation and transition-state theory, which are outlined as follows,
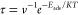
7where *T* is the temperature in K, we calculated the desorption time at normal body temperature (310 K); *v* is the attempt frequency (10^12^ Hz); and K represents the Boltzmann constant. The time required for desorption is exponentially related to the adsorption energy, indicating that a greater adsorption energy correlates with an extended desorption period. Temperature has a significant effect on the adsorption of the molecules. Adsorption is generally a temperature-dependent process, as described by thermodynamic principles. When the temperature increases, the kinetic energy of the molecules also increases, which can lead to enhanced desorption rates. This relationship can be understood through the van’t Hoff–Arrhenius equation, which relates the change in the equilibrium constant of the adsorption process to temperature. Higher temperatures can provide sufficient thermal energy to overcome the adsorption barrier, resulting in more molecules desorbing from the surface. Conversely, lower temperatures can favor adsorption by reducing the kinetic energy of the molecules. The interaction energies for Twin sheets are observed to be within a favorable range of −0.41 to −0.95 eV. At body temperature, the time taken for the desorption of 6-MP, GB, 5-FU, and CP from Twin-BN was recorded as 2782s, 2 ms, 4.62 μs, and 25 ms, respectively, while for Twin-Gr it took 8 ms, 2 ms, 9.78 μs, and 5 ms. The adsorption energy (*E*_ad_) of the complex molecules 6-MP, GB, 5-FU, and CP on Twin-BN are –0.95 eV ,–0.58 eV ,–0.41 eV, and −0.64 eV, respectively, while the corresponding values on Twin-Gr are −0.61 eV, −0.58 eV, −0.43 eV, and –0.60 eV. The calculated *E*_ad_ value for 6-MP, after adsorption on both Twin carriers, is higher compared to other ACD molecules. This implies a slower off-loading rate for 6-MP on both Twin-BN and Twin-Gr carriers. To address the issue of slow release, irradiation with visible light could prove to be beneficial. Numerous methods are utilized for the regulated liberation of medicinal substances, among which systems that initiate drug desorption through light have gained widespread adoption. Their prominence is attributed to the exceptional specificity and noninvasive characteristics they offer.^50^ Exposure to visible light can trigger a photothermal reaction, potentially enabling a precise release of ACD molecules at the site of the ailment. Moreover, the observed lower adsorption energy (*E*_ad_) for the other ACD molecules implies that GB, 5-FU, and CP are likely to be released more easily from the twin-boron nitride and twin-graphene surfaces at the intended site.

## 4 Conclusion

In this study, we explored the possibility of twin boron nitride and twin graphene monolayers as carriers for anticancer drugs (ACDs) by means of density functional theory (DFT) computations. We evaluated the interaction among these monolayers and four ACDs: 5-fluorouracil (5-FU), gemcitabine (GC), cyclophosphamide (CP), and mercaptopurine (6-MP). The adsorption of ACDs on the Twin-BN and Twin-Gr monolayers was found to be energetically favorable, indicating a strong interaction crucial for effective drug delivery. We determined the timeframes for drug release from the nanosheets by employing the van’t Hoff–Arrhenius equation and transition-state theory, which revealed that an increase in adsorption energy correlates with an extended duration of desorption. The desorption of 6-MP, GB, 5-FU, and CP from Twin-BN took 2782s, 2 ms, 4.62 μs, and 25 ms at body temperature, while for Twin-Gr it took 8 ms, 2 ms, 9.78 μs, and 5 ms. The calculated adsorption energy (Ead) value for 6-MP, after adsorption on both Twin carriers, is higher compared to those of other ACD molecules, implying a slower off-loading rate for 6-MP on both Twin-BN and Twin-Gr carriers. To address the issue of slow release, we suggest the use of irradiation with visible light, which could induce a photothermal effect and facilitate the controlled off-loading of ACD molecules at the disease site. However, the lower *E*_ad_ values observed for the other considered ACD molecules suggest that the GB, 5-FU, and CP molecules will offload more readily from Twin-BN and Twin-Gr at the targeted area. This study contributes to the advancement of innovative methodologies for drug administration, highlighting the potential of nanomaterials in this domain. Furthermore, our results will help experimental studies explore and validate these computational findings for the practical feasibility of using these monolayers as drug carriers.

## Data Availability

Data that support the findings of this study will be available from the corresponding authors upon reasonable request.

## Supporting Information Available

Electronic band structure plots, density of states plots, and table of calculated adsorption energies (PDF)

## References

[ref1] You L.; Lv Z.; Li C.; Ye W.; Zhou Y.; Jin J.; Han Q. Worldwide cancer statistics of adolescents and young adults in 2019: a systematic analysis of the Global Burden of Disease Study 2019. ESMO Open 2021, 6, 100255–100267. 10.1016/j.esmoop.2021.100255. 34481330 PMC8417345

[ref2] Ahmed T.; Rahman M. A.; Islam R.; Piya A. A.; Shamim S. U. D. Unravelling the adsorption performance of BN, AlN, GaN and InN 2D nanosheets towards the ciclopirox, 5-fluorouracil and nitrosourea for anticancer drug delivery motive: A DFT-D with QTAIM, PCM and COSMO investigations. Comput. Theor. Chem. 2022, 1214, 113797–113810. 10.1016/j.comptc.2022.113797.

[ref3] Munny K. N.; Ahmed T.; Piya A. A.; Shamim S. U. D. Exploring the adsorption performance of doped graphene quantum dots as anticancer drug carriers for cisplatin by DFT, PCM, and COSMO approaches. Struct. Chem. 2023, 34, 2089–2105. 10.1007/s11224-023-02150-y.

[ref4] Si W.; Shen J.; Zheng H.; Fan W. The role and mechanisms of action of microRNAs in cancer drug resistance. Clin. Epigenet. 2019, 11, 25–40. 10.1186/s13148-018-0587-8. PMC6371621 30744689

[ref5] Ren H.; Chen F.; Li X.; He Y. A new insight of structures, bonding and electronic properties for 6-mercaptopurine and Ag8 clusters configurations: a theoretical perspective. BMC Chem. 2019, 13, 55–65. 10.1186/s13065-019-0573-z. 31384803 PMC6661816

[ref6] Chen Z.; Zhang G.; Chen X.; Chen J.; Liu J.; Yuan H. A fluorescence switch sensor for 6-mercaptopurine detection based on gold nanoparticles stabilized by biomacromolecule. Biosens. Bioelectron. 2013, 41, 844–847. 10.1016/j.bios.2012.07.079. 22939508

[ref7] Nielsen O.; Vainer B.; Rask-Madsen J. The treatment of inflammatory bowel disease with 6-mercaptopurine or azathioprine. Aliment. Pharmacol. Ther. 2001, 15, 1699–1710. 10.1046/j.1365-2036.2001.01102.x. 11683683

[ref8] Vivoni A.; Chen S.-P.; Ejeh D.; Hosten C. M. Determination of the Orientation of 6-Mercaptopurine Adsorbed on a Silver Electrode by Surface-Enhanced Raman Spectroscopy and Normal Mode Calculations. Langmuir 2000, 16, 3310–3320. 10.1021/la9913194.

[ref9] Razmimanesh F.; Amjad-Iranagh S.; Modarress H. Molecular dynamics simulation study of chitosan and gemcitabine as a drug delivery system. J. Mol. Model. 2015, 21, 165–175. 10.1007/s00894-015-2705-2. 26044358

[ref10] Pfisterer J.; Plante M.; Vergote I.; du Bois A.; Hirte H.; Lacave A. J.; et al. Gemcitabine Plus Carboplatin Compared With Carboplatin in Patients With Platinum-Sensitive Recurrent Ovarian Cancer: An Intergroup Trial of the AGO-OVAR, the NCIC CTG, and the EORTC GCG. J. Clin. Oncol. 2006, 24, 4699–4710. 10.1200/JCO.2006.06.0913. 16966687

[ref11] Jia Y.; Xie J. Promising molecular mechanisms responsible for gemcitabine resistance in cancer. Genes Dis. 2015, 2, 299–307. 10.1016/j.gendis.2015.07.003. 30258872 PMC6150077

[ref12] Huang P.; Chubb S.; Hertel L. W.; Grindey G. B.; Plunkett W. Action of 2’,2’-Difluorodeoxycytidine on DNA Synthesis. Cancer Res. 1991, 51, 6110–6117. 1718594

[ref13] Gandhi V.; Plunkett W. Modulatory Activity of 2’,2’-Difluorodeoxycytidine on the Phosphorylation and Cytotoxicity of Arabinosyl Nucleosides. Cancer Res. 1990, 50, 3675–3681. 2340517

[ref14] Hertel L. W.; Boder G. B.; Kroin J. S.; Rinzel S. M.; Poore G. A.; Todd G. C.; et al. Evaluation of the Antitumor Activity of Gemcitabine (2’,2’-Difluoro-2’-deoxycytidine). Cancer Res. 1990, 50, 4417–4423. 2364394

[ref15] Isanbor C.; O’Hagan D. Fluorine in medicinal chemistry: A review of anti-cancer agents. J. Fluor. Chem. 2006, 127, 303–315. 10.1016/j.jfluchem.2006.01.011.

[ref16] Palasz A.; Ciez D. In search of uracil derivatives as bioactive agents. Uracils and fused uracils: Synthesis, biological activity and applications. Eur. J. Med. Chem. 2015, 97, 582–596. 10.1016/j.ejmech.2014.10.008. 25306174

[ref17] Lenz H.-J.; Stintzing S.; Loupakis F. TAS-102, a novel antitumor agent: A review of the mechanism of action. Cancer Treat. Rev. 2015, 41, 777–785. 10.1016/j.ctrv.2015.06.001. 26428513 PMC4624296

[ref18] Bormio Nunes J. H.; Bergamini F. R. G.; Lustri W. R.; de Paiva P. P.; Ruiz A. L. T. G.; de Carvalho J. E.; Corbi P. P. Synthesis, characterization and in vitro biological assays of a silver(I) complex with 5-fluorouracil: A strategy to overcome multidrug resistant tumor cells. J. Fluor. Chem. 2017, 195, 93–101. 10.1016/j.jfluchem.2017.01.016.

[ref19] Gel’fman M. I.; Kustova N. A. Instability constants of the complexes of Pt II, Pd II, Au I, and Ag I with 6-mercaptopurine. Russ. J. Inorg. Chem. 1969, 14, 110–111.

[ref20] Harahap Y.; Steven S.; Suryadi H. Development and validation of a UPLC-MS/MS method with volumetric absorptive microsampling to quantitate cyclophosphamide and 4-hydroxycyclophosphamide. Front. Pharmacol. 2022, 13, 928721 10.3389/fphar.2022.928721. 36034779 PMC9403605

[ref21] Felegari Z. Structural and Stability Investigation of the Anticancer Drug Cyclophosphamide via Quantum Chemical Calculations: A Nanotube Drug Delivery. Orient. J. Chem. 2014, 30, 1865–1872. 10.13005/ojc/300447.

[ref22] Wang S.; Meng Y.; Li C.; Qian M.; Huang R. Receptor-Mediated Drug Delivery Systems Targeting to Glioma. Nanomaterials 2016, 6, 3 10.3390/nano6010003. PMC5302535 28344260

[ref23] Luo M.; Yu Y.; Jin Z.; Dong H. L.; Li Y. Y. Multi-scale simulations on biocompatibility of boron nitride nanomaterials with different curvatures: A comparative study Appl. Surf. Sci. 2020, 517, 146181 10.1016/j.apsusc.2020.146181.

[ref24] Pal K.; Asthana N.; Aljabali A. A.; Bhardwaj S. K.; Kralj S.; Penkova A.; Thomas S.; Zaheer T.; Gomes de Souza F. A Critical Review on Multifunctional Smart Materials ‘Nanographene’ Emerging Avenue: Nanoimaging and Biosensor Applications. Crit. Rev. Solid State Mater. Sci. 2022, 47, 691–707. 10.1080/10408436.2021.1935717.

[ref25] Khorsand A.; Jamehbozorgi S.; Ghiasi R.; Rezvani M. Structural, Energetic and Electrical Properties of Encapsulation of Penicillamine Drug into the CNTs Based on vdW-DF Perspective. Physica E: Low-Dimensional Systems and Nanostructures 2015, 72, 120–127. 10.1016/j.physe.2015.04.015.

[ref26] Sabet M.; Tanreh S.; Khosravi A.; Astaraki M.; Rezvani M.; Darvish Ganji M. Theoretical Assessment of the Solvent Effect on the Functionalization of Au32 and C60 Nanocages with Fluorouracil Drug. Diam. Relat. Mater. 2022, 126, 109142 10.1016/j.diamond.2022.109142.

[ref27] Dabhi S.; Roondhe B.; Jha P. Nucleobases-Decorated Boron Nitride Nanoribbons for Electrochemical Biosensing: A Dispersion-Corrected DFT Study. Phys. Chem. Chem. Phys. 2018, 20, 8943–8952. 10.1039/C7CP08145F. 29557430

[ref28] Roondhe B.; Jha P. Haeckelite”, a New Low-Dimensional Cousin of Boron Nitride for Biosensing with Ultra-Fast Recovery Time: A First Principles Investigation. J. Mater. Chem. B 2018, 6, 6796–6803. 10.1039/C8TB01649F. 32254696

[ref29] Roondhe B.; Dabhi S.; Jha P. Sensing Properties of Pristine Boron Nitride Nanostructures towards Alkaloids: A First Principles Dispersion Corrected Study. Appl. Surf. Sci. 2018, 441, 588–594. 10.1016/j.apsusc.2018.01.249.

[ref30] Roondhe B.; Jha P. Neurotransmitter-Functionalized Boron Nitride Nanoribbons as Biological Cargo Carriers: Analysis by Density Functional Theory. ACS Appl. Nano Mater. 2019, 2, 1552–1561. 10.1021/acsanm.9b00028.

[ref31] Roondhe B.; Saha S.; Luo W.; Ahuja R.; Saxena S. Detection of type-II diabetes using graphene-based biosensors. J. Phys. D: Appl. Phys. 2024, 57, 185402 10.1088/1361-6463/ad2336.

[ref32] Jiang J.-W.; Leng J.; Li J.; Guo Z.; Chang T.; Guo X.; Zhang T. Twin graphene: A novel two-dimensional semiconducting carbon allotrope. Carbon 2017, 118, 370–375. 10.1016/j.carbon.2017.03.067.

[ref33] Kresse G.; Hafner J. Ab initio molecular dynamics for liquid metals. Phys. Rev. B 1993, 47, 558–561. 10.1103/PhysRevB.47.558. 10004490

[ref34] Perdew J. P.; Burke K.; Ernzerhof M. Generalized gradient approximation made simple. Phys. Rev. Lett. 1996, 77, 3865–3868. 10.1103/PhysRevLett.77.3865. 10062328

[ref35] Monkhorst H. J.; Pack J. D. Special points for Brillouin-zone integrations. Phys. Rev. B 1976, 13, 5188–5192. 10.1103/PhysRevB.13.5188.

[ref36] Yu M.; Trinkle D. R. Accurate and efficient algorithm for Bader charge integration. J. Chem. Phys. 2011, 134, 064111 10.1063/1.3553716. 21322665

[ref37] Grimme S. Semiempirical GGA-type density functional constructed with a long-range dispersion correction. J. Comput. Chem. 2006, 27, 1787–1799. 10.1002/jcc.20495. 16955487

[ref38] Kresse G.; Hafner J. Ab initio molecular dynamics for liquid metals. Phys. Rev. B 1993, 47, 558 10.1103/PhysRevB.47.558. 10004490

[ref39] Deb J.; Sarkar U. Boron-nitride and boron-phosphide doped twin-graphene: Applications in electronics and optoelectronics. Appl. Surf. Sci. 2021, 541, 148657 10.1016/j.apsusc.2020.148657.

[ref40] Zhang Y.; Yun J.; Wang K.; Chen X.; Yang Z.; Zhang Z.; Yan J.; Zhao W. First-principle study of graphyne-like BN sheet: Electronic structure and optical properties. Comput. Mater. Sci. 2017, 136, 12–18. 10.1016/j.commatsci.2017.04.006.

[ref41] Barman N.; Sarkar U. Twin graphene as an anode material for potassium-ion battery: A first principles study. Energy Storage 2024, 6, e673 10.1002/est2.673.

[ref42] Dua H.; Deb J.; Sarkar U. High capacitance twin-graphene anode material for magnesium ion battery. Energy Storage 2023, 5, e371 10.1002/est2.371.

[ref43] Majidi R.; Rabczuk T. Structural and electronic properties of BN co-doped and BN analogue of twin graphene sheets: A density functional theory study. J. Phys. Chem. Solids 2019, 135, 109115 10.1016/j.jpcs.2019.109115.

[ref44] Majidi R.; Ramazani A. Detection of HF and H2S with pristine and Ti-embedded twin graphene: A density functional theory study. J. Phys. Chem. Solids 2019, 132, 31–37. 10.1016/j.jpcs.2019.04.013.

[ref45] Roondhe B.; Ahuja R.; Luo W. Investigation of the adsorption behaviour of toxic heavy metals and bacteria on two allotropes of low dimensional boron nitride: Implications for detection and elimination. Appl. Surf. Sci. 2024, 667, 160404 10.1016/j.apsusc.2024.160404.

[ref46] Ehrenreich H.; Cohen M. H. Self-Consistent Field Approach to the Many-Electron Problem. Phys. Rev. 1959, 115, 786–789. 10.1103/PhysRev.115.786.

[ref47] Roondhe B.; Sanyal S. P.; Jha P. K.; Ahuja R.; Shukla S.; Saxena S. H- and T-Li2O monolayers: Latest addition to 2D flatlands. Appl. Surf. Sci. 2021, 556, 149737 10.1016/j.apsusc.2021.149737.

[ref48] Roondhe B.; Roondhe V.; Shukla A.; Shukla S.; Luo W.; Ahuja R.; Saxena S. Janus Functionalized Boron-Nitride Nanosystems as a Potential Application for Absorber Layer in Solar Cells. Adv. Electron. Mater. 2023, 9, 2300013 10.1002/aelm.202300013.

[ref49] Sajjad M.; Feng P. Study the gas sensing properties of boron nitride nanosheets. Mater. Res. Bull. 2014, 49, 35–42. 10.1016/j.materresbull.2013.08.019.

[ref50] Volodkin D. V.; Skirtach A. G.; Möhwald H. Near-IR remote release from assemblies of liposomes and nanoparticles. Angew. Chem., Int. Ed. 2009, 48, 1807–1810. 10.1002/anie.200805572. 19173270

