# Supplementary material for: Harnessing the Efficiency of Twin Boron Nitride and Graphene Monolayers for Anticancer Drug Delivery: Insights from DFT
Source: ACS Appl Bio Mater. 2025 Feb 7;8(3):2015–26. doi: 10.1021/acsabm.4c01507 (PMC11921028; doi:10.1021/acsabm.4c01507)
Supplement: Supplementary file 1 — mt4c01507_si_001.pdf [file mt4c01507_si_001.pdf]

Harnessing the Efficiency of Twin Boron Nitride and Graphene Monolayer for Anti-Cancer Drug Delivery: Insights from DFT

Basant Roondhe[1*], Rajeev Ahuja[1,2*] and Wei Luo[1*]

[1]*Condensed Matter Theory Group, Materials Theory Division, Department of Physics and Astronomy, Uppsala University, Box 516, Uppsala, 75120 Sweden.*

[2]*Department of Physics, Indian Institute of Technology Ropar, Rupnagar, Punjab, 140001 India.*

E-mail: basant.roondhe@physics.uu.se; rajeev.ahuja@physics.uu.se; wei.luo@physics.uu.se

Supporting Information

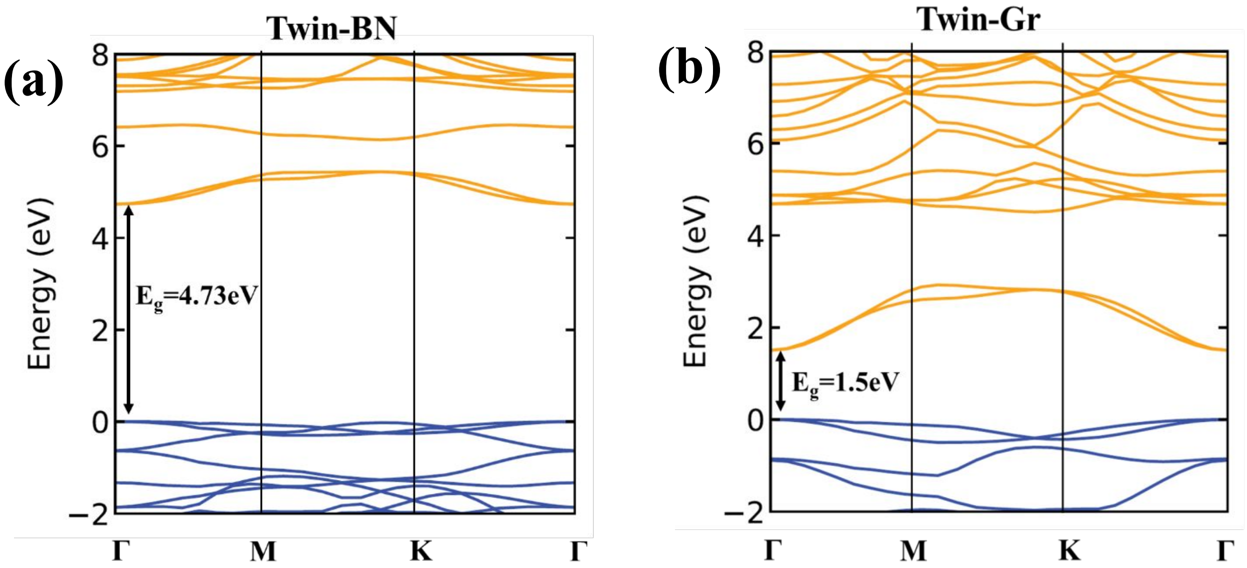

Figure S1: Electronic band structure plot of pristine calculated through HSE06 (a) Twin-BN and (b) Twin-Gr. The Fermi is set to 0eV.

Table S1: Calculated adsorption energy (E_{ad}) in (eV) with and without the incorporation of vdW correction through DFT-D3.

**System**	**With vdW (eV)**	**Without vdW (eV)**
**Twin-BN+5-FU**	-0.41	+0.01
**Twin-BN+GB**	-0.58	-0.01
**Twin-BN+CP**	-0.64	+0.02
**Twin-BN+6-MP**	-0.95	-0.17
**Twin-Gr+5-FU**	-0.43	+0.03
**Twin-Gr+GB**	-0.58	+0.02
**Twin-Gr+CP**	-0.60	+0.67
**Twin-Gr+6-MP**	-0.61	+0.15

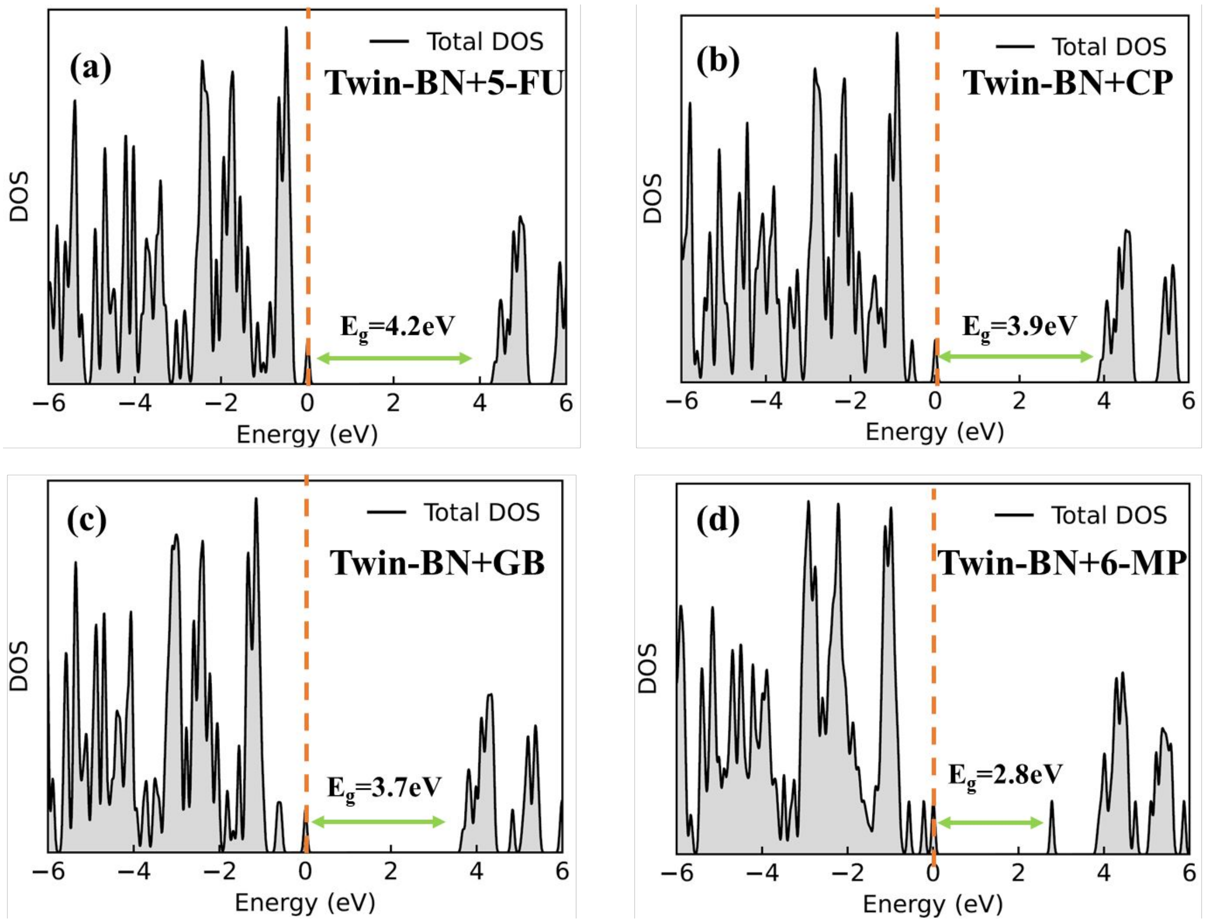

Figure S2: Density of states (DOS) plot of anticancer drugs adsorbed Twin-BN monolayer obtained with HSE06 (a) 5-FU@Twin- BN, (b) CP@Twin-BN, (c) GB@Twin-BN, (d) 6-MP@Twin-BN. The Fermi is set to 0eV.

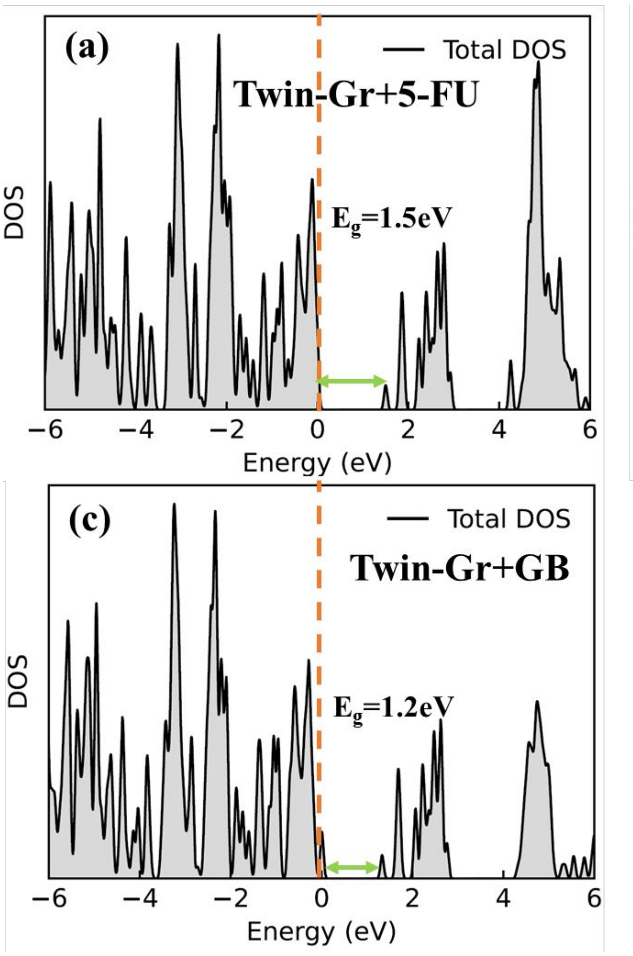

Figure S3: Density of states (DOS) plot of anticancer drugs adsorbed Twin-Gr monolayer obtained with HSE06 (a) 5-FU@Twin- BN, (b) CP@Twin-BN, (c) GB@Twin-BN, (d) 6-MP@Twin-BN. The Fermi is set to 0eV.
